# iASPP–PP1 complex is required for cytokinetic abscission by controlling CEP55 dephosphorylation

**DOI:** 10.1038/s41419-018-0561-6

**Published:** 2018-05-09

**Authors:** Kun Gao, Yuanyuan Zhang, Qing Shi, Jianong Zhang, Liang Zhang, Huiru Sun, Dongyue Jiao, Xiayin Zhao, Hongru Tao, Youheng Wei, Yuqi Wang, Hexige Saiyin, Shi-Min Zhao, Yao Li, Pingzhao Zhang, Chenji Wang

**Affiliations:** 1 Clinical and Translational Research Center, Shanghai First Maternity and Infant Hospital, Tongji University School of Medicine, Shanghai, China; 2 State Key Laboratory of Genetic Engineering, School of Life Sciences, Institute of Biomedical Sciences, Fudan University, Shanghai, China; 3 Drug Discovery and Design Center, State Key Laboratory of Drug Research, Shanghai Institute of Materia Medica, Chinese Academy of Sciences (CAS), Shanghai, China

## Abstract

Cytokinesis is the last step of cell division and is concluded by the abscission of the intercellular bridge that connects two daughter cells. The tight regulation of cytokinesis completion is essential because cytokinesis failure is associated with various human diseases. Here, we report that iASPP, a member of the apoptosis-stimulating proteins of p53 (ASPP) family, is required for proper cell division. iASPP depletion results in abnormal midbody structure and failed cytokinesis. We used protein affinity purification methods to identify the functional partners of iASPP. We found that iASPP associates with centrosomal protein of 55 kDa (CEP55), an important cytokinetic abscission regulator. Mechanically, iASPP acts as a PP1-targeting subunit to facilitate the interaction between PP1 and CEP55 and to remove PLK1-mediated Ser436 phosphorylation in CEP55 during late mitosis. The latter step is critical for the timely recruitment of CEP55 to the midbody. The present observations revealed a previously unrecognized function of iASPP in cytokinesis. This function, in turn, likely contributes to the roles of iASPP in tumor development and genetic diseases.

## Introduction

Cytokinesis is the final stage of cell division, and its completion results in the irreversible partitioning of a single eukaryotic cell into two daughter cells. Cytokinesis failure causes tetra- and polyploidization, which, in turn, can lead to genetic instability^1^. Similar to the other stages of cell division, cytokinesis is necessary for the proper growth and development of many organisms^2^. The deregulation of cytokinesis has been linked to various diseases, such as genetic disorders and cancers^2^.

Cytokinesis comprises several steps. The final stage of cytokinesis, termed abscission, requires the breakage of the midbody, a thin membranous stalk that connects nascent daughter cells. Cytokinetic abscission is a complex process that requires tight spatiotemporal regulation to ensure the equal distribution of genomic and cytoplasmic material between two nascent daughter cells^3^. Abscission, which involves membrane fission induced from the inside of the cell, is topologically similar to membrane fission during viral budding and multivesicular body formation^4^. The ESCRT-III membrane-remodeling complex is a key factor required by diverse membrane fission events^4^. Centrosomal protein of 55 kDa (CEP55) localizes in the midbody and plays crucial roles in cytokinesis^5,6^. CEP55 acts as an adaptor that interacts with the central MKLP-1 component of the midbody and ESCRT-I subunits TSG101 and ALIX, which recruit the ESCRT-III complex to cut the membrane link between newly formed daughter cells^7^.

iASPP, encoded by Protein Phosphatase 1 Regulatory subunit 13 Like (*PPP1R13L*), is evolutionarily conserved from worms to humans^8^. iASPP is a member of the Apoptosis Stimulating Proteins of p53 (ASPP) family proteins, which includes ASPP1 and ASPP2. iASPP inhibits, whereas ASPP1/2 stimulates, the proapoptotic activities of p53 (as well as family members p63 and p73)^9^. In addition to binding with p53, iASPP interacts with the NF-κB subunit p65RelA and inhibits its transcriptional activity^10^. iASPP strongly interacts with the catalytic subunits of protein phosphatase 1 (PP1) via a noncanonical motif (RNYF)^11^. iASPP likely acts as a regulatory subunit and targets the catalytic subunits of PP1 in specific subcellular compartments to selectively bind and dephosphorylate substrates; however, the exact substrates regulated by the iASPP–PP1 complex have not been reported^11^. Physiopathologically, iASPP is an important oncogene, and its expression is upregulated in various types of human cancers^12^. Moreover, *PPP1R13L* mutations in human, mice, or cattle all lead to a cardio–cutaneous syndrome associated with fatal dilated cardiomyopathy^13–15^. However, the molecular mechanism underlying these pathologies remains poorly understood.

We previously used tandem affinity purification (TAP) methods to reveal that ASPP1/2 is associated with a subset of kinetochore proteins^16^. Further studies demonstrated that ASPP1/2 are required for chromosome segregation and kinetochore–microtubule attachments^16^. In the present study, we showed that iASPP plays a critical role in cytokinetic abscission, the last step of cell division. Through TAP methods, we found that CEP55, a cytokinetic abscission regulator, is an interaction partner of iASPP. Moreover, we demonstrated that iASPP acts as a PP1-targeting subunit to facilitate the interaction between PP1 and CEP55. We also demonstrated that the iASPP–PP1 complex removes PLK1-mediated Ser436 in CEP55 during late mitosis. This step is critical for the timely recruitment of CEP55 to the midbody. Our study revealed that iASPP is a novel midbody-associated PP1 targeting subunit that plays critical roles in cytokinesis. This function might contribute to the tumor-promoting activity of iASPP.

## Results

### Identification of iASPP interactomes in HeLa cells

**Fig. 1:**
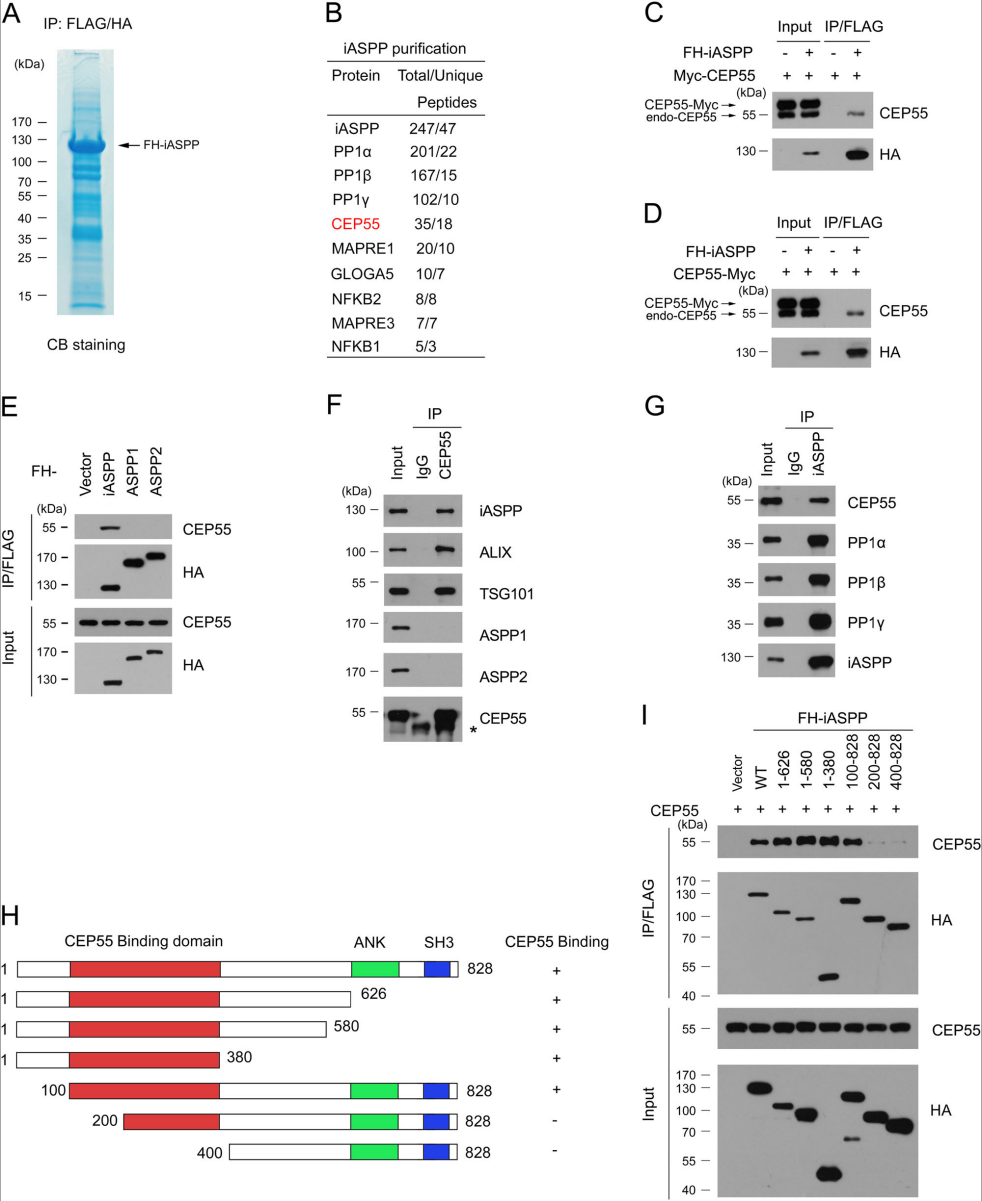
Identification of CEP55 as a novel iASPP interactor. **a**, **b** Tandem affinity purification of iASPP-containing protein complex was conducted using HeLa cells stably expressing FH-iASPP. Associated proteins were separated by SDS-PAGE and visualized by Coomassie Blue (CB) staining (**a**). The number of total/unique peptides identified by mass spectrometry analysis are shown in the table (**b**). **c**, **d** Exogenous overexpressed iASPP interacts with endogenous CEP55, but not the N-terminal (**c**) or C-terminal (**d**) Myc-tagged CEP55. 293T cells were co-transfected with indicated constructs. Cell lysates were prepared and subjected to immunoprecipitation with anti-FLAG antibody. The immunoprecipitates were analyzed by western blotting (WB) with indicated antibodies. **e** Exogenous overexpressed iASPP, but not the ASPP1/2, interacts with CEP55. 293T cells were transfected with indicated constructs. Cell lysates were prepared and subjected to immunoprecipitation with anti-FLAG antibody. The immunoprecipitates were analyzed by WB with indicated antibodies. **f**, **g** Endogenous iASPP interact with CEP55. Immunoprecipitation using anti-CEP55 (**f**) or iASPP (**g**) antibodies were performed using cell lysates prepared from HeLa cells. The immunoprecipitates was detected by WB with indicated antibodies. The asterisk (*) denotes a non-specific band. **h** Schematic representation of iASPP deletion mutants. Binding capacity of iASPP-WT or mutants to CEP55 is indicated with the symbols. **i** Identification of CEP55-binding domain in iASPP. 293T cells were transfected with indicated constructs. Cell lysates were prepared for immunoprecipitation with anti-FLAG antibody and analyzed by WB

To identify the molecular mediators of the cellular function of iASPP, we isolated the iASPP complex from HeLa cells stably expressing FLAG-HA-iASPP through TAP methods and determined the proteins present in the complex by using mass spectrometry (Fig. 1a, b; Supplementary Table. 1). HeLa cells were chosen for stable cell lines generation since these cell lines were frequently used in cell cycle study. As verification of the efficiency of this approach, the peptides of three PP1 catalytic subunits (PP1α, PP1β and PP1γ) were abundantly detected in the complex^11^. In addition to the known binding partners of iASPP, other proteins, such as cytokinesis proteins (CEP55), microtubule plus-end-tracking proteins (MAPRE1, MAPRE3), Golgi apparatus proteins (GLOGLA5), and NF-κB subunits proteins (NFKB1, NFKB2), involved in diverse biological processes were co-purified with the iASPP complex (Fig. 1b; Supplementary Table. 1). Given that the function of iASPP in cytokinesis has not been previously reported, we decided to further investigate the potential roles of iASPP in cytokinesis through its interaction with CEP55.

We first examined the interaction between iASPP and CEP55 by coexpressing FLAG–HA(FH)–iASPP and Myc-CEP55 in 293T cells. 293T cells were used for exogenous transfection since high efficiency transfection can be achieved in 293T cells. Cell lysates were subsequently prepared for coimmunoprecipitation (co-IP) with the anti-FLAG antibody. Interestingly, Myc–CEP55 was not immunoprecipitated by FH–iASPP, whereas endogenous CEP55 was immunoprecipitated by FH–iASPP (Fig. 1c). Similar co-IP results were obtained when we moved the Myc tag to the C-terminus of CEP55 (CEP55-Myc) (Fig. 1d). We also isolated the FH-CEP55 complex from HeLa cells through TAP methods and determined the proteins present in the complex, we found ALIX, TSG101 and other ESCRT-I subunits, were abundantly present in FH-CEP55 complex, However, we did not identify any peptides corresponding to iASPP (Suppl Table. 2). These results suggested that either the N- or C-terminal tag of CEP55 might hinder CEP55–iASPP interaction. The co-IP assay showed that ASPP1 and ASPP2, two other members of the ASPP family, do not interact with endogenous CEP55. This result suggested that the iASPP–CEP55 interaction is specific (Fig. 1e). Next, we decided to extend our analysis by investigating whether endogenous iASPP and CEP55 can interact with each other in HeLa cells. We performed immunoprecipitation with the anti-iASPP antibody by using cell lysates prepared from HeLa cells. As shown in Fig. 1f, CEP55 could immunoprecipitate iASPP and two known iASPP interactors (TSG101 and ALIX). By contrast, CEP55 was unable to immunoprecipitate iASPP paralogs ASPP1/2. A reciprocal co-IP assay also showed that endogenous iASPP interacts with CEP55 and three PP1 catalytic subunits (Fig. 1g). iASPP–CEP55 interaction were further verified in HCT116 p53(−/−) and A549 cells (Supp Fig. 1).

iASPP possesses an ankyrin domain (ANK) and a SH3 domain near its C-terminus (Fig. 1h). To identify the region that mediates the interaction of iASPP with CEP55, we performed co-IP assays with CEP55 and a series of iASPP deletion mutants in 293T cells. As shown in Fig. 1i, the region corresponding to the 100–200 aa of iASPP, which does not contain any known structural motifs, is responsible for CEP55 binding. This interaction pattern is different from the interaction pattern of other iASPP interactors, such as p53 and p65RelA, that bind to the C-terminal region (ANK–SH3 domain) of iASPP^8,10^.

### iASPP colocalizes with CEP55 at the midbody

**Fig. 2:**
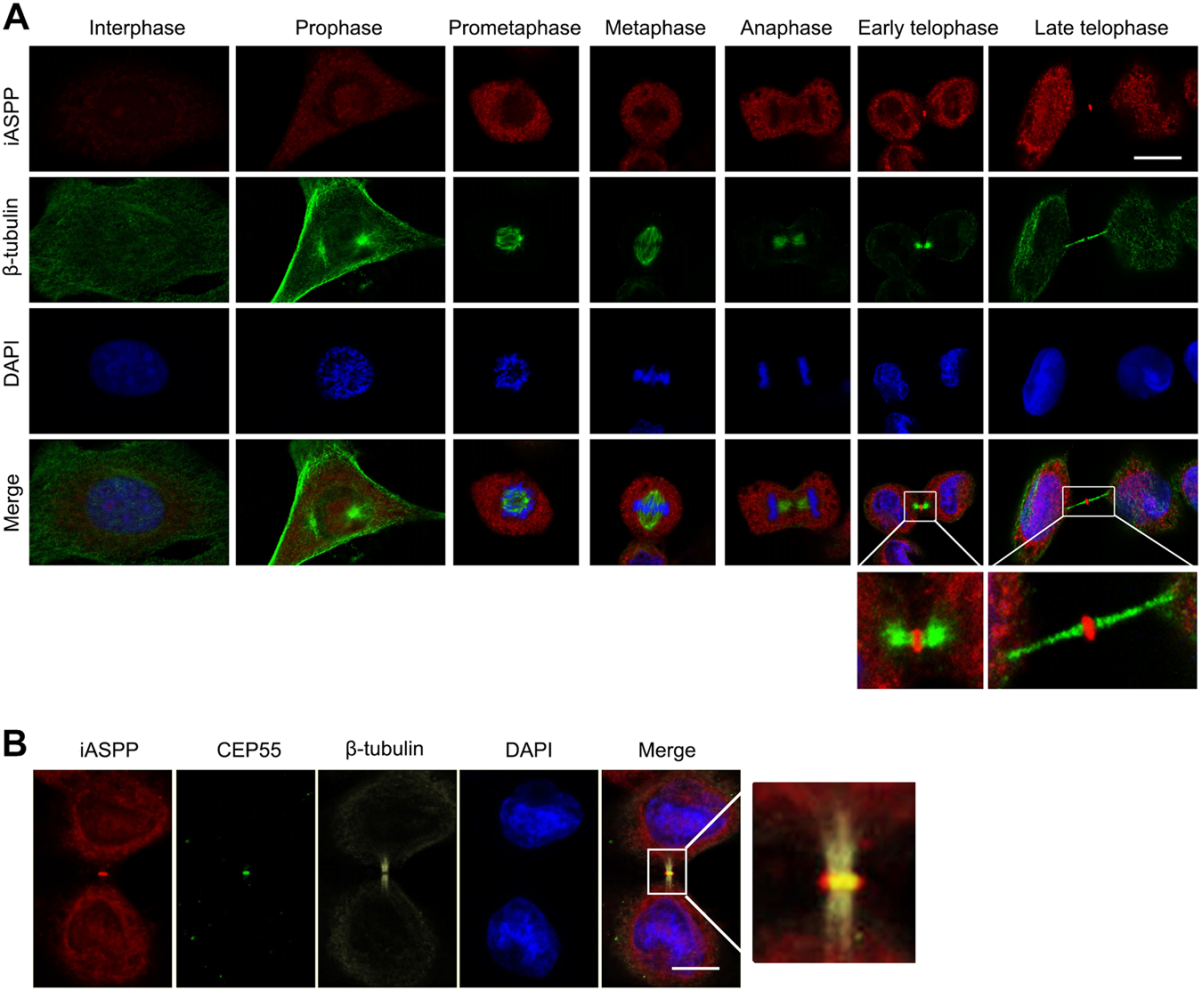
Cellular localization of endogenous iASPP during mitosis in human HeLa cells. **a** iASPP is detected at the midbody during cytokinesis. HeLa cells were immunostained for iASPP, β-tubulin, and DNA. The boxed areas in the merged images of cells in telophase showed at a higher magnification. Bar, 10 μm. **b** The colocalization of iASPP and CEP55 at midbody was detected by immunofluorescence. HeLa cells were immunostained for iASPP, β-tubulin, CEP55, and DNA

The fact that iASPP strongly interacts with the midbody protein CEP55 prompted us to examine the subcellular distribution of endogenous iASPP in HeLa cells at different phases of mitosis. As shown in Fig. 2a, iASPP localizes in the cytosol and nucleus during interphase. Interestingly, the protein did not accumulate during mitosis until telophase. During telophase, a small proportion of iASPP accumulates at the midbody (Fig. 2a), and colocalized with CEP55 at the midbody (Fig. 2b). Moreover, biochemical fraction of midbody and western blot (WB) analysis showed a small proportion of iASPP was present at midbody fraction (Supp Fig. 2).

### iASPP depletion causes cytokinesis defects in HeLa cells

**Fig. 3:**
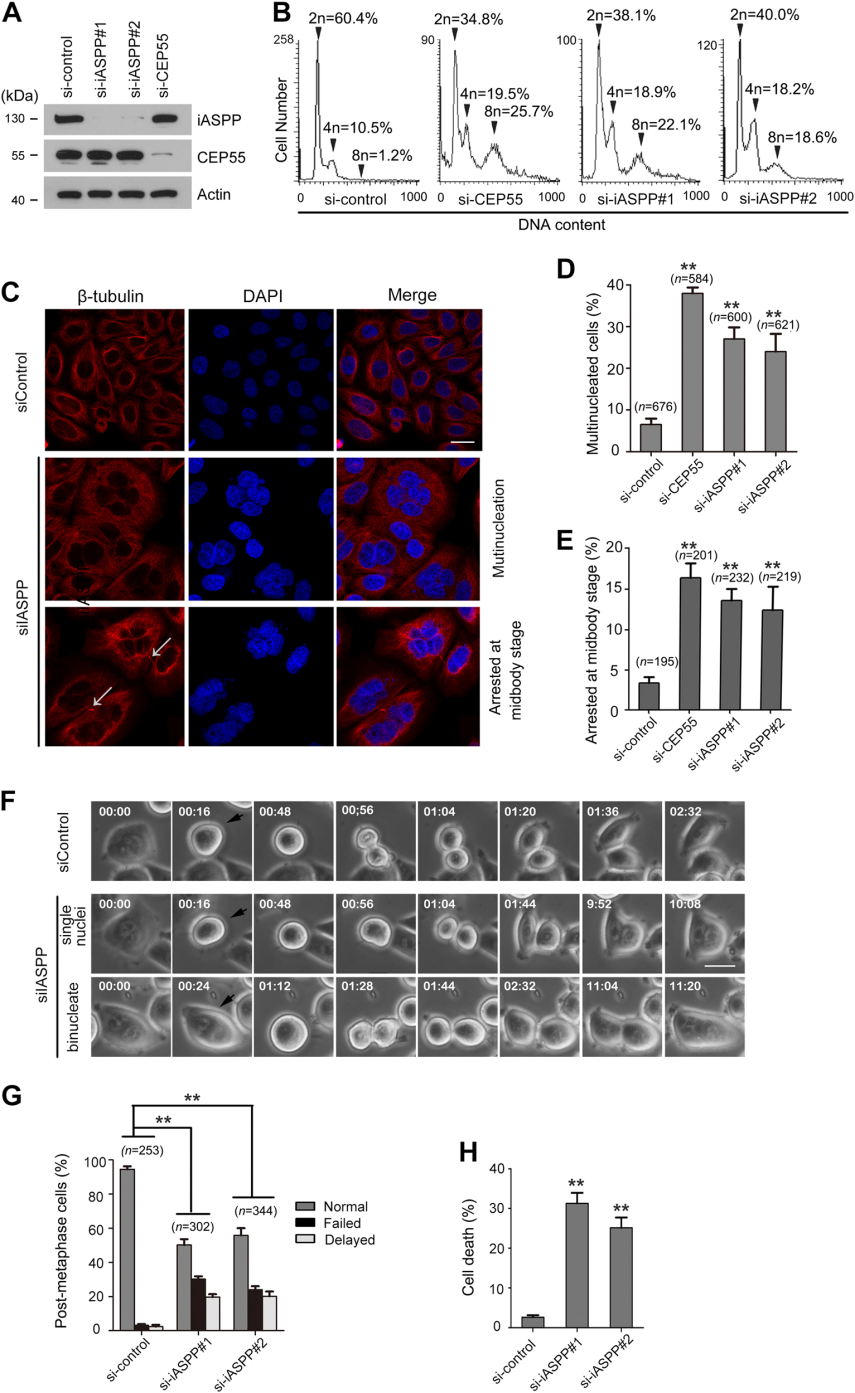
iASPP is required for the completion of cytokinesis. **a** Depletion of iASPP or CEP55 by siRNAs in HeLa cells. HeLa cells were transfected with indicated siRNAs. After 48 h, cell lysates were prepared for WB with indicated antibodies. **b** FACS analyses of the DNA content of cells depleted of iASPP, CEP55, or treated with control siRNAs. Peaks corresponding to 2n, 4n, and 8n DNA contents are labeled, and the cell percentage volumes are provided. **c** HeLa cells transfected with the indicated siRNAs were stained with β-tubulin antibodies (red) and DAPI (blue). **d**, **e** Quantification of multinucleated cells (**d**) and cells arrested at the midbody (**e**) in control, iASPP-depleted, or CEP55-depleted HeLa cells. All data shown are mean values ± SD (error bar) from three replicates. **p < 0.01, Student’s *t*-test. **f** Representative time-lapse phase-contrast images of a control cell proceeding through mitosis is shown (upper panel). In the lower panels, representative time-lapse images of iASPP-depleted cells with either one or two nuclei proceeding through mitosis are shown. Black arrowheads highlight cells that are starting to divide. **g** Quantitation of post-metaphase HeLa cells treated with control or iASPP siRNAs. All data shown are mean values ± SD (error bar) from three replicates. **p < 0.01, Student’s *t*-test. **h** iASPP depletion leads to increased cell death. HeLa cells were transfected with control or iASPP siRNAs. After 72 h, the cell death was measured by flow cytometry using the propidium iodide staining assay. All data shown are mean values ± SD (error bar) from three replicates. **p < 0.01, Student’s *t*-test

To test whether iASPP is required for cytokinesis, we examined cell division in iASPP-depleted HeLa cells. Cells transfected with CEP55-targeting siRNAs were used as the positive control to monitor abscission defects. WB analyses confirmed that at 48 h after siRNA transfection, the iASPP or CEP55 protein levels of iASPP-depleted cells decreased to less than 10% of those of control cells (Fig. 3a). We then performed FACS analyses to measure the relative numbers of HeLa cells with 2n, 4n, and 8n DNA contents. The frequencies of iASPP-depleted cells with 4n DNA contents markedly increased by approximately two-fold of those of control cells with 4n DNA contents (Fig. 3b). Moreover, the frequencies of iASPP-depleted cells with 8n DNA contents dramatically increased by 15-fold of those of control cells with 8n DNA contents (Fig. 3b). These profiles indicated that the cytokinesis failure leads to the accumulation of cells with multiple nuclei and multiple DNA copies.

Microscopy analysis further confirmed that iASPP-depleted HeLa cells are highly aberrant, with most of the cells exhibiting multiple nuclei (Fig. 3c, middle panel) and/or unusual cytokinetic bridges that appeared to be the remnants of arrested midbodies (Fig. 3c, lower panel). By contrast, no significant cytokinesis defects were observed in control cells (Fig. 3c, upper panel). Quantification showed that iASPP depletion by two independent siRNAs markedly increased the percentage of multinucleated cells (from 6.5 to 24.0% or 27.1%) and cells arrested at midbody (from 3.3 to 13.3% or 13.5%) (Fig. 3d, e). These phenotypes were similar to those exhibited by CEP55-depleted cells (Fig. 3b, d, e), further supporting previous reports that CEP55 has a critical function in cytokinesis^5,6^. We also observed iASPP depletion in HCT116 p53(−/−) and A549 cells also caused cytokinesis defects, suggesting these phenotypes were not specific to Hela cells (Supp Fig. 3A-H)

To further characterize the phenotypes associated with failed cytokinesis, we subjected live HeLa cells transfected with control or iASPP siRNAs to time-lapse microscopy analysis. The cell division duration was measured from the onset of roundup to when the two cells clearly separated. The majority of control siRNA-transfected cells that entered mitosis underwent normal cytokinetic abscission to form two daughter cells with normal kinetics (2 h 15 min ± 5 min; Fig. 3f). iASPP-depleted cells also progressed through cell roundup to telophase with normal kinetics (56 ± 6 min; Fig. 3f). However, ~50% of iASPP-depleted cells displayed delayed failed cytokinesis (Fig. 3g). Consistent with our observations on fixed cells, these live cells remained in post-telophase stage and remained connected by a thin cytokinetic bridge for a prolonged period of time. Moreover, their division duration increased by up to 10 h prior to the regression of the cleavage furrow; these effects resulted in the formation of a binucleated cell (Fig. 3f).

Aberrant and incomplete mitosis often leads to mitotic catastrophe, a form of cell death^17^. Accordingly, PI staining assay showed that the rates of cell death markedly increased following prolonged iASPP depletion (Fig. 3h). Mitotic catastrophe precedes and uses antiproliferative measures, including apoptosis or necrosis, to prevent the proliferation of defective mitotic cells^18^. We also observed that cell apoptosis rates, as measured through Annexin V staining assay, markedly increased following prolonged iASPP depletion (Supp Fig. 3I). In summary, these results suggested that iASPP is required for proper cytokinesis.

### iASPP is required for CEP55 recruitment to the midbody during cytokinesis

**Fig. 4:**
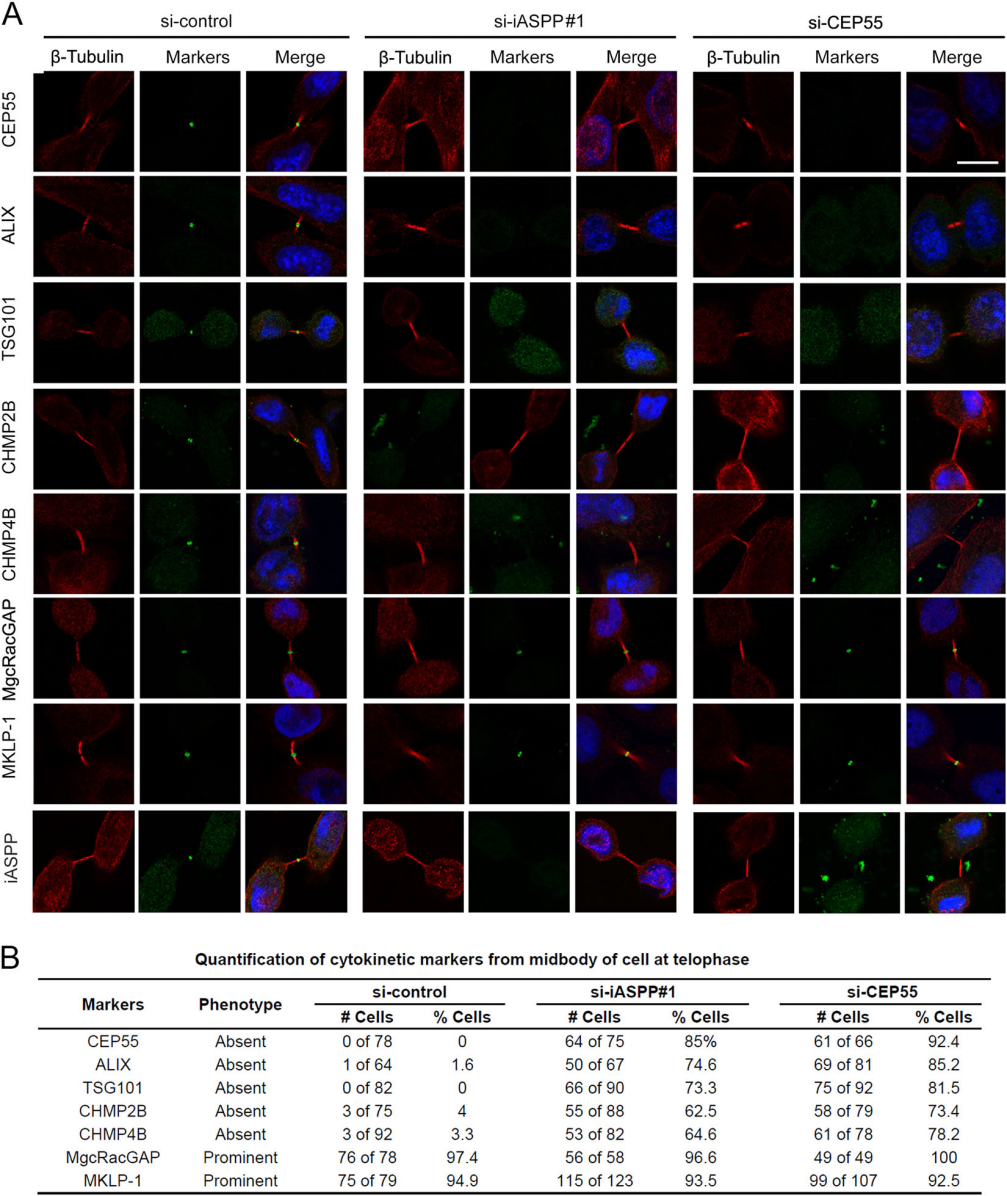
iASPP-dependent localization of midbody markers. **a** Localization of midbody markers in control, iASPP-depleted, or CEP55-depleted HeLa cells. HeLa cells were transfected with indicated siRNAs. After 48 h, cells were immunostained for midbody markers (green), β-tubulin (red), and DNA (blue). **b** Quantification of proportions of cells from **a** displaying defects in localization of markers at the midbody

The aberrant recruitment of CEP55 to the midbody results in abscission failure^5,6^. Given that iASPP depletion did not alter CEP55 protein levels (Fig. 3a), we investigated whether iASPP depletion affects the recruitment of CEP55 to the midbody during telophase. Immunofluorescence analysis revealed that upon iASPP depletion, CEP55 was absent from the midbody in the majority of cells (Fig. 4a, b). Given that CEP55 is required for the recruitment of ESCRT-I and ESCRT-III components to the midbody, we next investigated whether the recruitment of ESCRT-I and ESCRT-III components to the midbody is similarly affected by iASPP depletion. As expected, ESCRT-I components (TSG101 and ALIX) and ESCRT-III components (CHMP2B and CHMP4B) were absent from the midbody in the majority of iASPP-depleted cells (Fig. 4a, b). To ensure that the recruitment defects observed upon iASPP depletion are caused by CEP55 misregulation, but not general defects in midbody formation, we investigated the midbody localization of MKLP-1 and MgcRacGAP, which are two components of the centralspindalin complex recruited before CEP55 to the midbody^5^. As shown in Fig. 4a, b, iASPP depletion did not affect the localization of MKLP-1 or MgcRacGAP at the midbody. Biochemical fraction of midbody proteins and WB analysis also demonstrated that CEP55 was absent from the midbody fraction in iASPP-depleted HeLa cells. (Supp Fig. 3). Taken together, these results suggested that iASPP positively regulates cytokinesis by prompting CEP55 recruitment to the midbody.

### iASPP facilitates the interaction between CEP55 and PP1γ

We next attempted to explore the molecular mechanism that underlies the iASPP-controlled localization of CEP55 to the midbody. Previous reports showed that PLK1 phosphorylates CEP55 at Ser436 and prevents its premature recruitment to the anaphase spindle^19^. A PLK1 phosphorylation site mutant of CEP55 (S436A) that is prematurely recruited to the anaphase spindle fails to support abscission^19^. During late mitosis, the PLK1-mediated CEP55 phosphorylation must be removed for the timely recruitment of CEP55 to the midbody; the molecular mechanism that underlies this process, however, is largely unknown^19^. We hypothesized that iASPP functions as a midbody-associated PP1 targeting subunit that antagonizes PLK1-mediated CEP55 S436 phosphorylation during late mitosis, a process that may be critical for the midbody recruitment of CEP55.

**Fig. 5:**
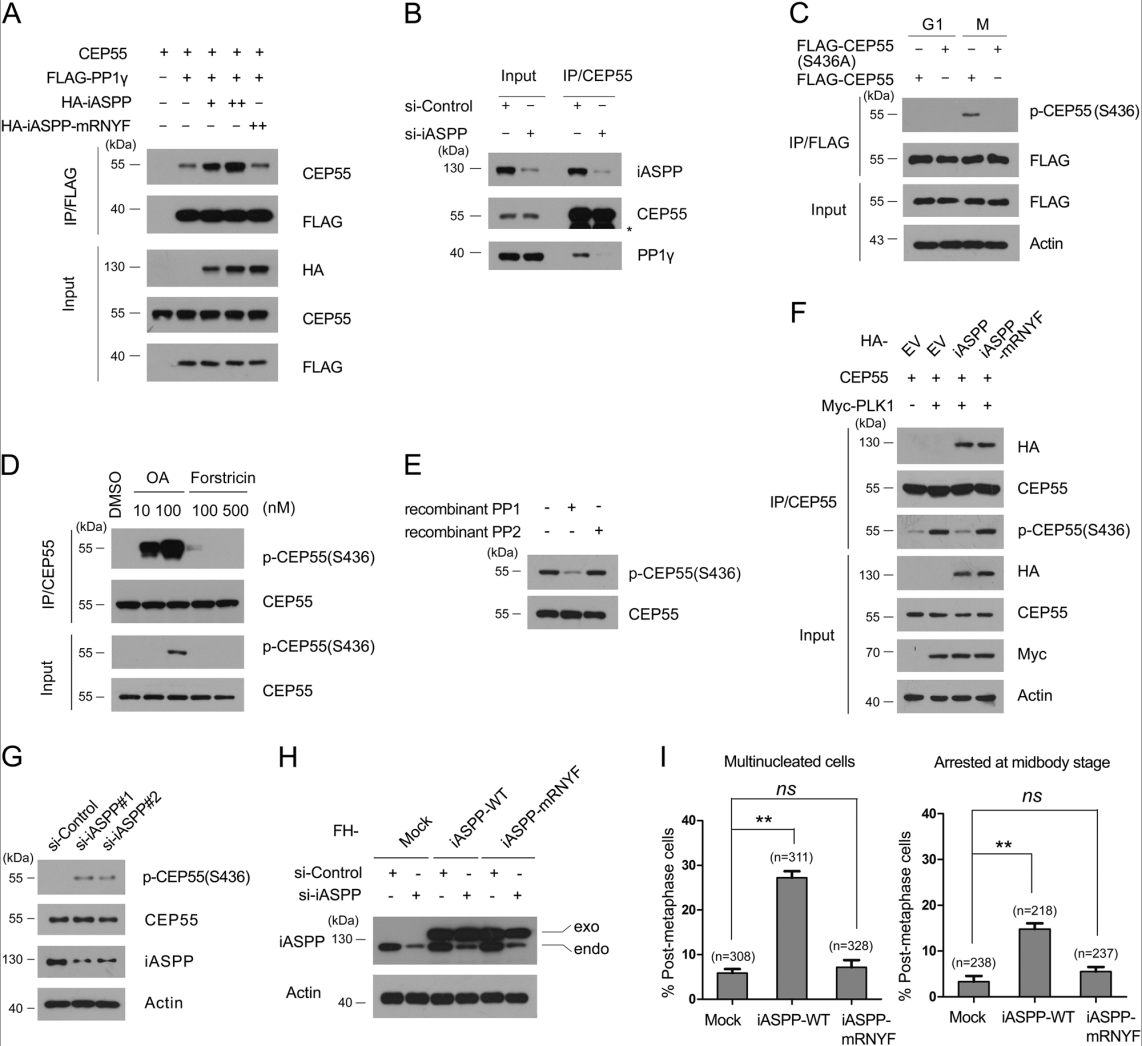
iASPP–PP1 complex dephosphorylates mitotic CEP55 at Ser436. **a** iASPP facilitates the interaction between CEP55 and PP1γ in a PP1-binding-dependent manner. 293T cells were co-transfected with indicated constructs. After 24 h, cell lysates were prepared for immunoprecipitation with anti-FLAG antibody and WB analyses using indicated antibodies. **b** iASPP depletion reduces the endogenous interaction between CEP55 and PP1γ. HeLa cells were transfected with control or iASPP siRNAs. After 48 h, cell lysates were prepared for immunoprecipitation with anti-CEP55 antibody and WB analyses using indicated antibodies. The asterisk (*) denotes a non-specific band. **c** Validation of phospho-CEP55 (S436) antibody. HeLa cells were transfected with FLAG-CEP55 or CEP55 S435A mutant constructs. After 48 h, cell lysates were prepared for immunoprecipitation with anti-FLAG antibody and WB analyses using indicated antibodies. **d** PP1, but not the PP2 inhibitor increases phospho-CEP55 (S436) signal. HeLa cells were treated with different doses of Okadaic acid (OA) or Fostriecin for 12 h. Cell lysates were prepared for immunoprecipitation with the anti-CEP55 antibody and detected by WB analyses using the indicated antibodies. **e** PP1 dephosphorylates CEP55 at Ser436 in vitro. HeLa cells were arrested in prometaphase by a sequential thymidine-nocodazole block, then endogenous CEP55 was immunoprecipitated and incubated with the recombinant PP1 or PP2. The phospho-CEP55 (S436) signal was analyzed by WB using the indicated antibodies. **f** iASPP antagonizes PLK1-mediated CEP55 (S436) phosphorylation. 293T cells were transfected with pcDNA3-CEP55、FLAG-PP1γ and different dose of HA-iASPP (WT or mRNYF) constructs. After 24 h, cell lysates were prepared for immunoprecipitation with anti-FLAG antibody and WB using indicated antibodies. **g** iASPP depletion increases phospho-CEP55 (S436) signal. HeLa cells were transfected with control or iASPP siRNA. After 48 h, cell lysates were prepared for WB analyses with indicated antibodies. **h** WB analyses of iASPP proteins following siRNA treatment in HeLa cells stably expressing a FH-iASPP constructs resistant to the siRNAs targeting endogenous iASPP. **i** Stably expression of siRNA-resistant iASPP, but not mRNYF, in HeLa cells rescued cytokinesis defects caused by iASPP depletion. All data shown are mean values ± SD (error bar) from three replicates

Our previous study showed that ASPP1/2 can facilitate the interaction between Hec1 and PP1α to promote Hec1 dephosphorylation during late mitosis^16^. Similarly, we investigated whether iASPP can act as a molecular adaptor that facilitates the interaction between CEP55 and PP1γ. We selected PP1γ in subsequent studies because this PP1 subunit localizes in the midbody and is required for the completion of cytokinesis^20^. As expected, the co-IP assay showed that the co-expression of iASPP markedly increased the interaction between CEP55 and PP1γ in a dose-dependent manner (Fig. 5a). iASPP has a noncanonical PP1-binding motif (RNYF) that is located within its SH3 domain^11^. To test whether the interaction of iASPP with PP1γ is required for iASPP to enhance CEP55–PP1γ interaction, we generated an iASPP mRNYF mutant that carries four substitutions in each of its conserved motifs (RNYF–AAAA). As expected, the iASPP mRNYF mutant lost its ability to enhance CEP55–PP1γ interaction (Fig. 5a). In agreement with the above findings, iASPP depletion significantly reduced the interaction between CEP55 and PP1γ (Fig. 5b). In summary, these results suggested that iASPP can facilitate the interaction between CEP55 and PP1γ in a PP1-binding-dependent manner.

### iASPP–PP1 complexes dephosphorylate mitotic CEP55 at S436

Given that iASPP facilitates the interaction between CEP55 and PP1γ, we next investigated whether iASPP modulates the mitotic phosphorylation of CEP55. We generated a phospho-CEP55 (S436) antibody that specifically recognizes ectopically expressed wild-type CEP55 but not the CEP55 S436A mutant during mitosis (Fig. 5c). Treatment with Okadalic acid (PP1 inhibitor) but not with Fostriecin (PP2A inhibitor) greatly enhanced the phospho-CEP55 (S436) signal. This result suggested that dephosphorylation at CEP55 (S436) is regulated by PP1 but not by PP2A (Fig. 5d). We also performed an in vitro dephosphorylation assay and found that recombinant PP1 but not PP2 weakened the phospho-CEP55 (S436) signal (Fig. 5e). To test whether iASPP acts as a PP1-targeting subunit to regulate the dephosphorylation of CEP55 phospho-S436, iASPP (WT or mRNYF), CEP55 and PLK1 constructs were co-expressed in 293T cells. CEP55 was immunoprecipitated, and the phospho-CEP55 (S436) signal was detected. As shown in Fig. 5f, co-expression with iASPP-WT but not with the iASPP-mRNYF mutant markedly reduced PLK1-mediated CEP55 (S436) phosphorylation. Therefore, these results suggested that iASPP can antagonize PLK1-mediated CEP55 (S165) phosphorylation in a PP1-binding-dependent manner. Moreover, iASPP depletion in HeLa cells intensified the basal phospho-CEP55 (S436) signal (Fig. 5g).

To further investigate whether iASPP-mediated cytokinesis is dependent on PP1-binding capability, we generated HeLa cells stably expressing siRNA-resistant iASPP (WT or mRNYF) (Fig. 5h). When endogenous iASPP was depleted by siRNAs, significant rescue of cytokinesis defects were observed in HeLa cells stably expressing iASPP-WT but not in HeLa cells stably expressing the iASPP-mRNYF mutant (Fig. 5i). In conclusion, our results suggested that iASPP promotes the dephosphorylation of CEP55, at least at S436, during late mitosis. Moreover, the PP1 binding ability of iASPP is indispensable for its function in cell division.

## Discussion

In this study, we revealed that iASPP has an unexpected function as a midbody-associated PP1 targeting subunit in cytokinesis. We also demonstrated that CEP55 is a substrate that is dephosphorylated by the iASPP–PP1 complex during late mitosis. Entry into and progression through mitosis are driven by the phosphorylation of thousands of substrates under the master control of various mitotic kinases, such as CDK1 and PLK1^21^. However, phosphatase activities rise to cooperate with the declining activities of mitotic kinases to reset the phosphorylation status of proteins to the general low level that is characteristic of G1-phase cells^22^. Studies on yeast and human cells have revealed a timely defined dephosphorylation program that coordinates and regulates events in anaphase, telophase, and cytokinesis^23–25^. PP1 and PP2A have been proposed to be the two main phosphatases that contribute to mitotic substrate dephosphorylation and mitotic exit progression in animal cells^25^. In vivo, PP1 specificity is mainly achieved through the association of a catalytic subunit with specific targeting subunits that drive localization and modulate activity and specificity^26^. Our previous studies demonstrated that ASPP1/2 are required for proper mitotic progression. However, in contrast to iASPP depletion, ASPP1/2 depletion does not lead to cytokinesis defects in HeLa cells. Instead, ASPP1/2 codepletion causes G2/M cell cycle arrest, defective kinetochore–microtubule attachments, and persistent spindle assembly checkpoint activation^16^. We revealed that ASPP1/2 acts as PP1-targeting subunits that facilitate the interaction of PP1 with kinetochore protein Hec1 and catalyze Hec1 (S165) dephosphorylation during late mitosis^16^. Moreover, we found that the ASPP1/2–PP1 complex dephosphorylates C-Nap1 (S2417/2421) to regulate centrosome linker reassembly during late mitosis^27^. We propose that the ASPP family of proteins is a class of PP1-targeting subunits with functions in late mitosis. Our findings expand the repertoire of PP1-targeting subunits, including KNL-1, Sds22, Repo-Man, and MYPT1, that function in mitotic exit^26^.

Whether the iASPP–PP1 complex could dephosphorylate other interactors during mitotic exit remains unclear. Through TAP–MS, we identified golgin84 (GLOLGA5) as an iASPP–PP1 interaction candidate (Supplementary Table 1). Golgin84 is a coiled-coil protein located at the cytoplasmic surface of the Golgi apparatus. It has been implicated in the maintenance of the structural integrity of the Golgi apparatus by acting as a tethering factor for retrograde vesicle transport^28^. During prophase, the Golgi apparatus undergoes extensive fragmentation through a multistage process to allow for its correct partitioning and inheritance by daughter cells. The fragmented Golgi apparatus is reassembled during telophase. The reassembly process is dependent on the mitotic phosphorylation and post-mitotic dephosphorylation of multiple Golgi-associated proteins^29^. Interestingly, Golgin-84 is highly phosphorylated during mitosis^28^. Our preliminary results confirmed that iASPP interacts with Golgin-84. Moreover, Golgi reassembly was greatly compromised in iASPP-depleted cells (data not shown). Thus, future studies should investigate whether the iASPP–PP1 complex dephosphorylates Golgi proteins to facilitate mitotic exit.

In addition to S436, CEP55 S425, and S428 are phosphorylated by ERK2 and CDK1 upon mitotic entry. PLK1 binds to the phosphorylation sites generated by ERK2 and CDK1 to mediate the S436 phosphorylation of CEP55^6^. The present study did not investigate the involvement of the iASPP–PP1 complex in ERK2 removal and CDK1-mediated CEP55 S425/428 phosphorylation during late mitosis because of the lack of phospho-S425/428 antibodies. However, future studies will address this topic. Moreover, we noticed that another phosphatase complex, PP4 (PPP4C/PPP4R1), was co-purified with the CEP55 protein complex (Supplementary Table. 2). The involvement of PP4 or other unidentified phosphatases in CEP55 dephosphorylation during late mitosis is an interesting topic that requires further study.

The tumor-promoting activity of iASPP has been attributed to p53 inhibition. However, p53-independent activities exist given that iASPP is expressed in various p53-null cell lines^30^. Indeed we observed iASPP depletion in p53 null HCT116 p53(−/−) cells also caused cytokinesis defects, suggesting iASPP’s role in cytokinesis is p53-independent (Supp Fig. 3). A growing body of evidence suggests that the inactivation or hyperactivation of cytokinesis regulators causes cytokinesis failure, which results in tetra- and polyploidization^1^. Tumors derived from tetraploid/polyploidy cells exhibit progressive structural and numerical chromosomal instability, a hallmark of the majority of solid human cancers^1^. iASPP and CEP55 expression frequently occurs in tumors and are usually correlated with poor clinical prognosis^12,31^. It is still unclear whether iASPP overexpression enhances tumorigenesis and progression, at least in part, by promoting tetraploid/polyploidy. Although iASPP play a critical role in cytokinesis in cellular models, iASPP null mice is still viable but lead to a cardio–cutaneous syndrome associated with fatal dilated cardiomyopathy. It might be possible that the iASPP-mediated CEP55 dephosphorylation could be compensated by other phosphatases, such as PP4, during embryonic development. However, iASPP-regulated cytokinesis might be important for some adult organs such as heart, since iASPP showed highest expression level in heat tissues. Previous study reported that a truncating mutation in CEP55 cause human MARCH syndrome affecting neuronal mitosis (a high incidence of multinucleated neurons)^32^. Further studies on cells, mouse models, and patient tissue samples are required to investigate whether the deregulation of iASPP-regulated cytokinesis is important to tumorigenesis and developmental disorders.

## Materials and methods

### Cell culture and manipulations

293T, HeLa, A549, and HCT116 p53(−/−) cells were obtained from the American Type Culture Collection (ATCC). All cells were maintained in Dulbeco’s Modified Eagle Medium (DMEM) with 10% (v/v) fetal bovine serum (FBS). All cells were grown at 37 °C with 5% CO_2_. 293T cells are generally used for exogenous transfection. Endogenous Co-IP and functional experiments are done in Hela, HCT116 p53(−/−) or A549 cells.

### Expression constructs

The ASPP1, iASPP, PP1γ, and CEP55 cDNAs were obtained from Genecopoeia Inc. The ASPP2 cDNAs was kindly provided by Dr. Xin Lu (University of Oxford). All the cDNAs were subcloned into pCIN4-FLAG–HA (FH) and pCMV-Myc/HA vectors. CEP55 cDNAs were also subcloned into pCDNA3.0 vectors to express CEP55 protein without any tag. iASPP-mRNYF constructs were generated by the KOD-Plus Mutagenesis Kit (TOYOBO). To generate the RNAi-resistant FH-iASPP (WT or mRNYF mutant) construct, the wobble codons corresponding to the siRNA oligos of iASPP was mutated using KOD-Plus Mutagenesis Kit.

### RNA interference and rescue

For siRNA treatments, cells were transfected using Lipofectamine RNAiMAX (Invitrogen) and 0.05 μM siRNA oligos. The siRNA oligos were purchased from Genepharma Inc: si-iASPP #1 (GGGACUUUCUGGACAUGAATT), si-iASPP#2 (CCCUCAGCAUGAUCUUCAATT). To rescue the defects caused by iASPP depletion, stable cells lines that express RNAi-resistant FH-iASPP similar with endogenous iASPP levels were used for siRNA treatments.

### Protein complex purification

The epitope-tagging strategy to isolate iASPP or CEP55-containing protein complexes from human cells was performed essentially as previously described with some modifications^44^. In brief, to obtain a FLAG-HA-iASPP (or CEP55) expressing cell line, HeLa cells were transfected with pCIN4-FLAG-HA-iASPP (or CEP55) constructs and selected for 2 weeks in 1 mg/ml G418. The tagged iASPP protein levels were detected by WB analyses. The stable cell lines were chosen to expand for protein complex purification. For purification, the cells were lysed in BC100 buffer (20 mM Tris-Cl, pH 7.9, 100 mM NaCl, 0.2 mM EDTA, 20% glycerol) containing 0.2% Triton X-100 and fresh protease inhibitor on ice for 2 h. The homogenate was centrifuged for 30 min at 12,000 rpm at 4 °C. Cleared lysates were filtered through 0.45 μM spin filters (Millipore) and immunoprecipitated by anti-FLAG antibody-conjugated M2 agarose (Sigma). The bound polypeptides eluted with the FLAG peptide (Sigma) were further affinity purified by anti-HA antibody-conjugated agarose (Sigma). The final elutes from the HA-beads with HA peptides were resolved by SDS-PAGE on a 4–20% gradient gel (Bio-Rad) for Coomassie Blue staining. Gel bands were cut out from the gel and subjected to mass-spectrometric sequencing.

### Mass-spectrometric sequencing

Nano-LC MS/MS experiment was performed on an HPLC system composed by two LC-20AD nano-flow LC pumps, an SIL-20 AC auto-sampler and an LC-20AB micro-flow LC pump(Shimadzu) connected to an LTQ-Orbitrap mass spectrometer (Thermo Fisher). Sample was loaded onto a CAPTRAP column (0.5 x 2 mm, MICHROM Bioresources) in 5 min at a flow rate of 10 μl/min. The sample was subsequently separated by a C18 reverse-phase column (0.075 × 150 mm, packed with 3 μm Aeris C18 particles, Phenomenex) at a flow rate of 300 nl/min. The mobile phases were 0.1% formic acid as the loading phase and 4% acetonitrile in 0.1% formic acid (phase A) and 96% acetonitrile with 0.1% formic acid (phase B). To achieve proper separation, a 60-min linear gradient from 5 to 40% phase B was employed. The separated sample was introduced into the mass spectrometer via nanoelectrospray. The spray voltage was set at 2.3 kV and the heated capillary at 180 °C. The mass spectrometer was operated in data-dependent mode and each cycle of duty consisted one full-MS survey scan at the mass range 300–1600 Da with resolution power of 100,000 using the Orbitrap section, followed by MS/MS experiments for 10 strongest peaks using the LTQ section. The AGC expectation during full-MS and MS/MS were 1,000,000 and 10,000, respectively. Peptides were fragmented in the LTQ section using collision-induced dissociation with helium and the normalized collision energy value set at 35%. Only 2+ and 3+ peaks were selected for MS/MS run and previously fragmented peptides were excluded for 60s. Protein searches were performed with the Mascot 2.3.02 software (MatrixScience) against the SWISSPROT human protein database (release 2014_07) with the following criteria: 2 possible missed cleavage sites with enzymeset to trypsin, peptide mass tolerance of 25 ppm, fragment mass tolerance of 0.80 Da, Acetylated protein N-term and oxidized Met were considered as variable modifications. The acceptance criterion for peptide identifications was the rate of false positive identification less than 1%.

### Immunoprecipitation and western blotting

To immunoprecipitate the ectopically expressed FLAG-tagged proteins, transfected cells were lysed 24 h after transfection in FLAG-lysis buffer. The cell lysates were immunoprecipitated with the anti-FLAG antibody-conjugated M2 agarose beads at 4 °C overnight. After three washes with FLAG-lysis buffer, followed by two washes with BC100 buffer, the bound proteins were eluted using FLAG-Peptide/BC100 for 3 h at 4 °C. The eluted material was resolved by SDS-PAGE. For western blotting, cell lysates or immunoprecipitates were subjected to SDS-PAGE and proteins were transferred to nitrocellulose membranes. The membrane was blocked in Tris-buffered saline (TBS, pH 7.4) containing 5% non-fat milk and 0.1% Tween-20, washed twice in TBS containing 0.1% Tween-20, and incubated with primary antibody for 2 h and followed by secondary antibody for 1 h at room temperature. Afterward, the proteins of interest were visualized using ECL chemiluminescence system (Santa Cruz). Midbodies were isolated from thymidine/nocodazole-synchronized HeLa cells as described previously^33^.

### Antibodies

Antibody specific to phospho-CEP55 (S436) was raised against the synthetic phosphopeptide CKRIFKDLGTPFAL(pS)KSSM. Phospho-specific antibody was obtained though two-step affinity-purification methods. Commercially available antibodies for WB were as follows: ASPP1 (ab137537; Abcam), ASPP2 (611354; BD Biosciences), iASPP (18590-1-AP; Proteintech), CEP55 (23891-1-AP; Proteintech), CEP55 (7825-1; epitomics), α-Tubulin (1878-s; epitomics), β-Tubulin (05–661; Millipore), ALIX (sc-67338; Santa Cruz), TSG101 (4A10; Genetex), CHMP2B (12527-1-AP; Proteintech), CHMP4B(13683-1-AP; Proteintech), MKLP-1 (sc-867; Santa Cruz), MgcRacGAP (H00029127; Novus), PP1α (1950–1; epitomics), PP1β (2029–1; epitomics), PP1γ (6646–1; epitomics), Myc (9E10; Sigma), FLAG (M2; Sigma), HA (MM5-101R; Millipore), and Actin (AC-74; Sigma).

### Flow cytometry analysis

Flow cytometry analysis was performed and analyzed by flow cytometry (FACSCalibur, BD Biosciences) following cell DNA staining with propidium iodide (PI, Sigma). Briefly, 1×10^6^ cells were harvested and suspended with ice-cold 70% ethanol (in distilled water), then fixed at −20 °C for at least 2 h. Following harvesting and washing, cells were stained with 0.5 ml of propidium iodide (10 µg/ml) and RNase (100 µg/ml) in PBS for 30 min at room temperature in the dark and then submitted to flow cytometry analysis. For cell apoptosis analysis, cells were harvested and washed, followed by propidium iodide staining (10 µg/ml) with 0.03% triton permeation and RNase treatment. Results are representative of three independent experiments with triplicate samples for each condition.

### In vitro phosphatase assay

The purified PP1 and PP2 enzymes were purchased from Upstate (PP1/PP2A Toolbox, Catalog#17–301). HeLa cells were arrested in prometaphase by a double-thymidine block. Then they were lysed with lysis buffer, and the cell lysates were prepared and subjected to immunoprecipitation with anti-CEP55 antibody. The immunoprecipitates were divided equally, and 0.5 unit of PP1 or PP2 was added. After incubation for 30 min at 30 °C, the cell lysates were added to SDS–PAGE. The phospho-CEP55 (S436) signal was analyzed by WB.

### Immunofluorescence, confocal microscopy, and time-lapse microscopy

For immunofluorescence, cells were plated on chamber slides, fixed either with methanol at −20 °C for 5 min or with 4% paraformaldehyde at 37 °C for 15 min depending on the antibodies used. To examine the protein levels at each mitotic stage, cells were synchronized by double-thymidine block and release to fresh media for various times. A staging system was used to identify the different phases of mitosis and cytokinesis based on the DNA and spindle morphology and extent of chromosome alignment and separation. To test the stability of Microtubule capture at kinetochores, cells were incubated for 5 min on ice before fixation, to destabilize most non-kinetochore Microtubules. After fixation, cells were permeabilized with 0.2% Triton for 5 min, preincubated with centrifuged (14,000 rpm) supernatant of 5% FBS and 5% goat serum in PBS and incubated with primary antibodies overnight. Slides were washed, incubated with fluorescence-tagged secondary antibodies (Alexa Fluor 488, 568 and 647, Molecular probes, Invitrogen), and counterstained with DAPI (Vector Labs) for 1 h at 4 °C. Cells were visualized and imaged using a Zeiss LSM710 confocal microscope equipped with a ×60 objective. All immunofluorescence experiments were conducted at least three times. Image processing and figures were made using PhotoShop CS (Adobe). Images of proteins of interest as well as CREST on kinetochores were acquired by using identical imaging settings. For quantifying kinetochore intensities using Image J, a circular region with fixed diameter was centered over the kinetochore and intensities were measured for both the protein of interest and for CREST. CREST was used for normalization after subtraction of background intensity. Experiments were carried out with three or more replicates unless otherwise stated. Statistical analyses were performed by Student’s *t-*test for most studies. Values with *p < 0.01 are considered statistically significant.

### Time-lapse microscopy analysis

siRNA-transfected cells were cultured in a 37 °C microscope chamber with 5% CO_2_. Cells were viewed with a Zeiss Cell Observer microscope. The time-lapse series was acquired on a Cell Observer system consisting of a fully motorized Axiovert 200M microscope, ×20 objective, Axiocam HRm and AxioVision 3.1 using the Time-lapse modules. Imaging was performed for 24 h with a lapse time of 8 min.

## Electronic supplementary material

Figure. S1

Figure. S2

Supplementary Table

Supplementary figure legends

Figure. S3
