# Supplementary figures and images for: iASPP–PP1 complex is required for cytokinetic abscission by controlling CEP55 dephosphorylation
Source: Cell Death Dis. 2018 May 9;9(5):528. doi: 10.1038/s41419-018-0561-6 (PMC5943338; doi:10.1038/s41419-018-0561-6)

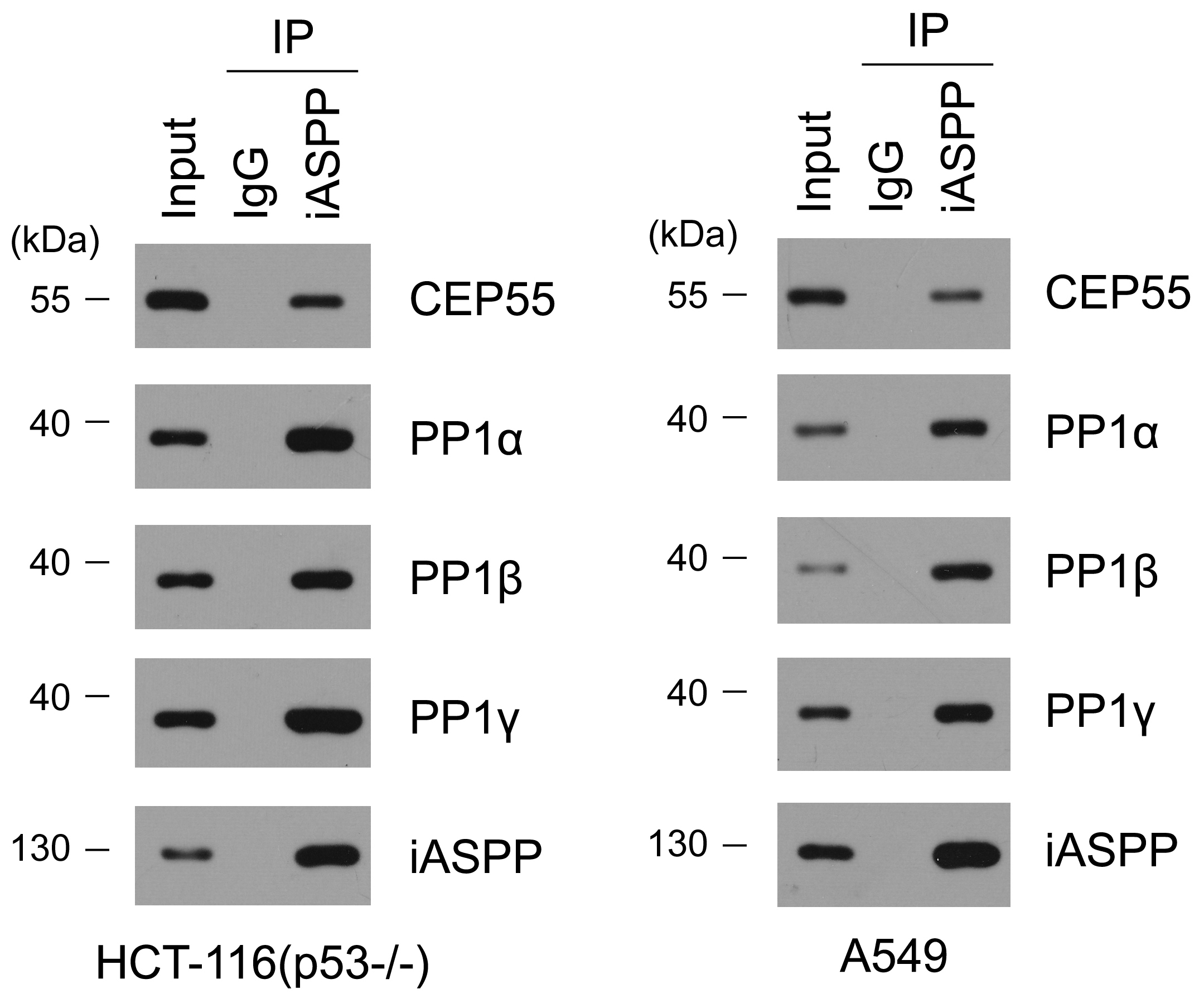

Supplement: Supplementary file 1 — Figure. S1 [file 41419_2018_561_MOESM1_ESM.jpg]

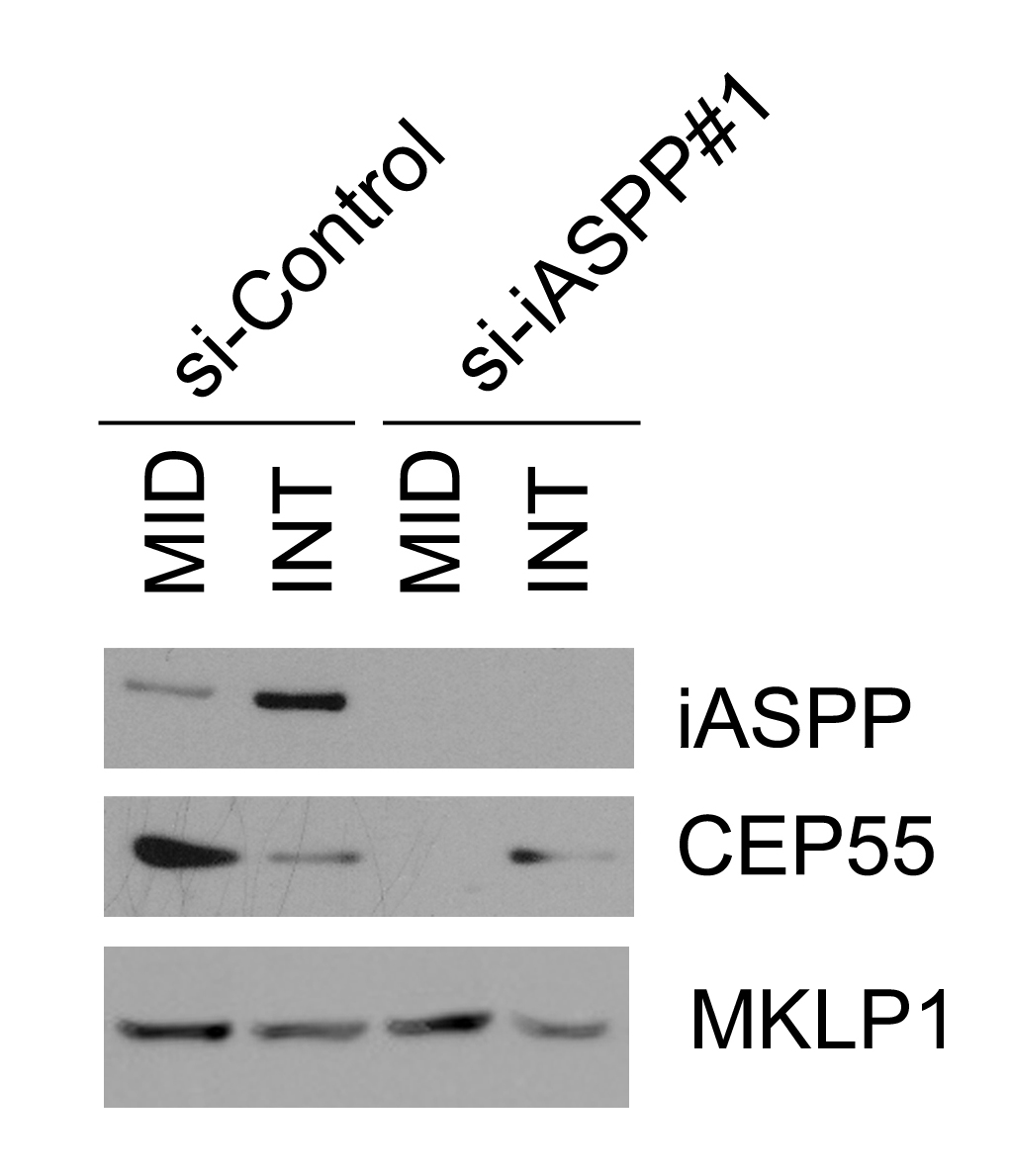

Supplement: Supplementary file 2 — Figure. S2 [file 41419_2018_561_MOESM2_ESM.jpg]

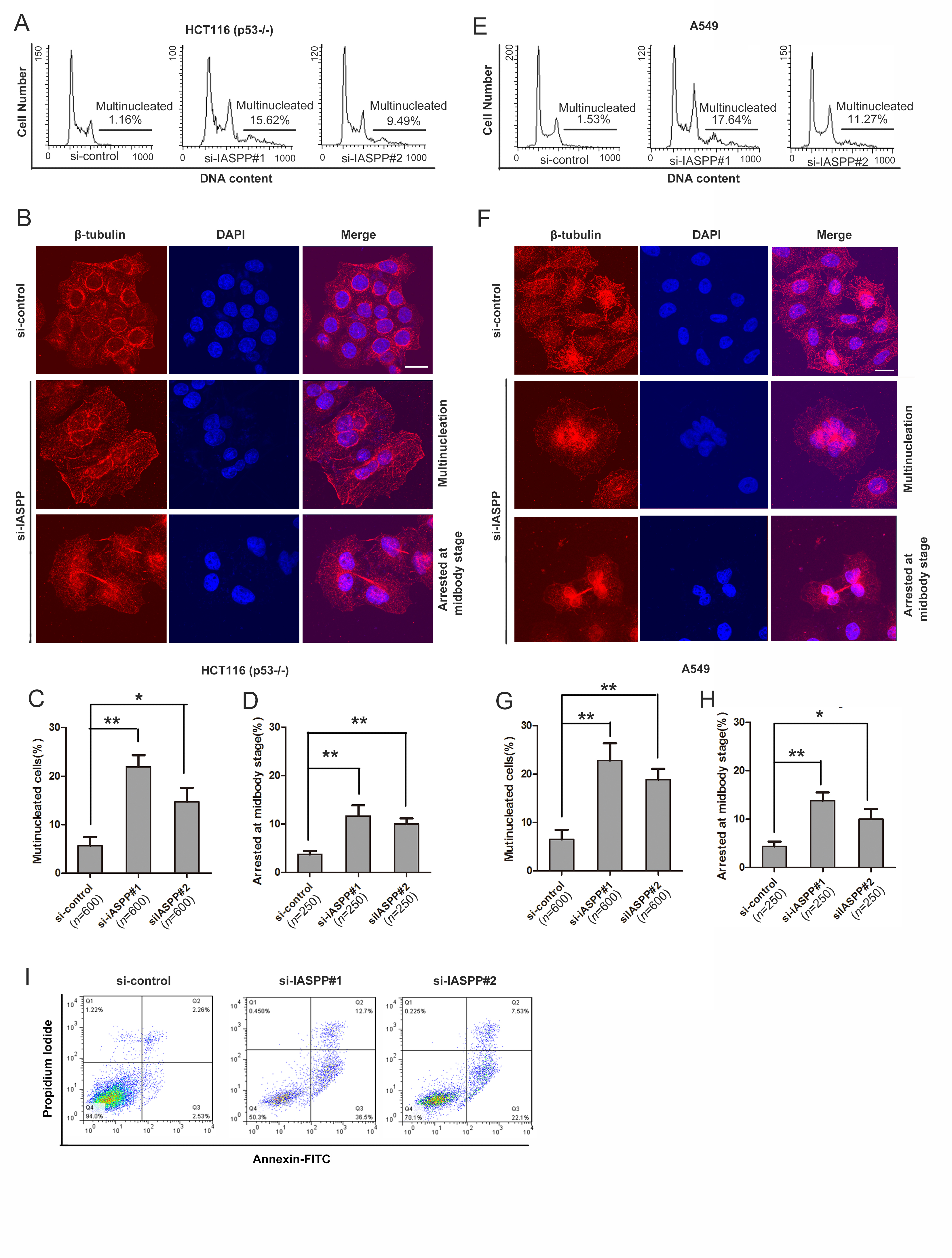

Supplement: Supplementary file 5 — Figure. S3 [file 41419_2018_561_MOESM5_ESM.jpg]
