# Supplementary material for: iASPP–PP1 complex is required for cytokinetic abscission by controlling CEP55 dephosphorylation
Source: Cell Death Dis. 2018 May 9;9(5):528. doi: 10.1038/s41419-018-0561-6 (PMC5943338; doi:10.1038/s41419-018-0561-6)
Supplement: Supplementary file 4 — Supplementary figure legends [file 41419_2018_561_MOESM4_ESM.docx]

**Figure S1. Endogenous iASPP interact with CEP55 in HCT-116(p53-/-) and A549 cells.** Immunoprecipitation using anti- iASPP antibody were performed using cell lysates prepared from HCT-116(p53-/-) or A549 cells. The immunoprecipitates was analyzed by WB with indicated antibodies.

**Figure S2. iASPP is required for CEP55 recruitment to the midbody (related to Figure 4).** HeLa cells were transfected with the indicated siRNAs. Midbodies (MID) fraction isolated from synchronized HeLa cells and fraction prepared from unsynchronized interphase cells (INT) were analyzed by WB with indicated antibodies.

**Figure S3. iASPP is required for the completion of cytokinesis (related to Figure 3).**

(A) FACS analyses of the DNA content of cells depleted of iASPP or treated with control siRNAs in HCT116 p53(-/-) cells. (B) HCT116 p53(-/-) cells transfected with the indicated siRNAs were stained with β-tubulin antibodies (red) and DAPI (blue). (C, D) Quantification of multinucleated cells (C) and cells arrested at the midbody (D）in control or iASPP-depleted HCT116 p53(-/-) cells. All data shown are mean values ± SD (error bar) from three replicates. ** p<0.01, Student’s *t*-test. (E) FACS analyses of the DNA content of cells depleted of iASPP or treated with control siRNAs in A549 cells. (F) A549 cells transfected with the indicated siRNAs were stained with β-tubulin antibodies (red) and DAPI (blue). (G, H) Quantification of multinucleated cells (G) and cells arrested at the midbody (H）in control or iASPP-depleted A549 cells. All data shown are mean values ± SD (error bar) from three replicates. ** p<0.01, Student’s t-test. (I) HeLa cells were transfected with control or iASPP siRNAs. After 72hr, the apoptosis was measured by flow cytometry using the Annexin V staining assay.
